# Morbidity and psychomotor development of offspring of women with gestational diabetes: a 5-year follow-up

**DOI:** 10.1186/s12887-022-03543-4

**Published:** 2022-08-20

**Authors:** Vendula Bartáková, Beáta Barátová, Katarína Chalásová, Petr Janků, Kateřina Kaňková

**Affiliations:** 1 Department of Pathophysiology, Faculty of Medicine, Masaryk University, Kamenice 5, Brno, 625 00 Czech Republic; 2 Department of Pediatrics, University Hospital Brno, Černopolní 9, Brno, Czech Republic; 3 Department of Obstetrics and Gynaecology, University Hospital Brno, Jihlavská 20, 625 00 Brno, Czech Republic

**Keywords:** Gestational diabetes mellitus, Obesity, Offspring, Pregnancy, Prospective study

## Abstract

**Background:**

Gestational diabetes mellitus (GDM) represents a risk factor for both mother and her offspring in a short-term (perinatal morbidity) and long-term horizon (postpartum diabetes or foetal programming). Several studies focused at peri/postnatal outcomes of GDM mother´s offspring, however relatively few (and none in Czech population) were designed as prospective. The aim of the study was to ascertain eventual anthropometric and developmental abnormalities and/or morbidity in offspring of GDM mothers compare to controls in a 5-year follow-up using a parent-reported parameters related to psychomotor development and common paediatric morbidities including a sub-study of offspring of GDM mothers experiencing adverse perinatal outcomes.

**Methods:**

A 5 year follow up study of offspring of GDM mothers (*n* = 26) vs those with a normal pregnancy (*n* = 63). An electronic questionnaire was used to obtain the parameters (such as growth, psychomotor development, vaccination, morbidity history etc.) available to parents from the parent-held infant health record. Data on pregnancy and delivery were available from the previous study.

**Results:**

Offspring of GDM mothers had delayed psychomotor development in early childhood, but in 5 years of age they seemed to gradually achieve results of a control group. Children with macrosomia had a higher percentile of weight-for-height and were significantly more frequently ill than those with a normal birth weight. Offspring of obese mothers had worse verbal language skills in early childhood and a higher percentile of weight-for-height.

**Conclusion:**

Maternal gestational diabetes and obesity can be considered an important determinant of postnatal offspring development and health status, which further advocates for broader implementation of preventive strategies.

## Background

Gestational diabetes mellitus (GDM) is a common complication of pregnancy whose incidence is rising worldwide for multiple reasons – a problem of overweight and obesity pertains to more and more younger women, babies are born to older mothers, and also screening programs contribute to increasing cumulative prevalence, no matter what criteria are being used for diagnostics [1]. GDM is defined as any degree of glucose intolerance first diagnosed in pregnant women (most often during the period of 24^th^ to 28^th^ week of gestation by a compulsory oral glucose tolerance test [oGTT]), that usually disappears after delivery [2].

Women with GDM have an increased risk of not only adverse perinatal outcomes (i.e. during pregnancy up to 1 year postpartum) such as hypertension or preeclampsia, complications during delivery, difficult breastfeeding etc., but also of persistence of abnormalities in glucose metabolism postpartum. They confer 7 × higher likehood to develop glucose abnormality (prediabetes or type 2 diabetes mellitus (T2DM)) in future life [3, 4]. Moreover, GDM represents a significant risk for the offspring increasing the incidence of macrosomia, hyperbilirubinemia, hypoglycaemia, or respiratory distress syndrome during delivery. Children of GDM mothers are also more susceptible to suffer from cardiovascular diseases (CVD) and childhood obesity or to develop T2DM more frequently in subsequent life [5].

Evidence is emerging that a substantial part of diabetes susceptibility is acquired early in life, probably owing to exposure to high glucose and certain hormonal stimulation in utero (the phenomenon of “foetal programming”) via epigenetic programming [6]. The evidence that GDM or pre-gestational overt T2DM in pregnancy can affect diabetes risk in offspring is limited but suggestive [7].

Obesity plays a significant role in the GDM development with some studies indicating worse parturition and offspring development in obese mothers independently of GDM (as elegantly summarized in a meta-analysis of Sachnez et al. [8]) and even more frequent adverse outcomes in offspring of obese plus GDM mothers [9].

Majority of previous studies dealing with both maternal and offspring morbidities focused on type 1 diabetes mellitus (T1DM) or T2DM mothers, only a few retrospective studies focused on GDM. Abnormal maternal glucose tolerance during pregnancy was independently associated with offspring higher body mass index (BMI) and overweight risk from 1 to 6 years of age [10]. A systematic review aimed at the incidence of birth defects showed the association between GDM and congenital heart defects and neural tube defects but only in women with both GDM and pre-pregnancy obesity [11]. Furthermore, very few prospective studies targeted children of GDM mothers. The largest one—American HAPO-FUS study—comprised more than 4000 children of GDM mothers and examined associations of maternal glycaemia during pregnancy with childhood glucose metabolism [12] and adiposity [13].

In a meta-analysis comprising 6140 infants, maternal diabetes (type 1 or 2) has been proposed to negatively affect the cognitive abilities of the child [14]. A small retrospective Polish study with 59 children found no abnormalities in a psychomotor development in offspring of GDM mothers or healthy mothers [15].

Based of thorough search we identified only one prospective study, 25 years old, focusing on psychomotor development in a cohort of offspring of diabetic mothers comprising a total of 196 subject of which 101 had GDM [16]. Recently, we analysed our own study cohort of *n* = 432 pregnant women (364 GDM cases and 68 controls) for perinatal morbidity [17] with the aim to assess the quality of prenatal GDM care in Czech Republic. Our results showed minimal differences in selected outcomes between GDM and control group at the time of delivery and overall excellent diabetes control in GDM group. As a continuation of these findings documenting a very satisfactory prenatal care and GDM management in our country we therefore intended to investigate whether eventual differences in the postnatal development of offspring of (well controlled) GDM mothers compared to controls exist at all since this is a relatively understudied area and recent management of GDM changed considerably. Furthermore, there has not been a similar study performed on Czech or other central European population reporting offspring data with a current standard of GDM care and according to our notion no similar studies were performed or being conducted at present. The specific objectives of the current study were to (i) ascertain possible anthropometric and psychomotor development abnormalities and/or morbidity in offspring of GDM mothers compare to non-diabetic pregnant controls during a 5-year follow-up and to (ii) describe the later consequences of documented perinatal morbidity (adverse perinatal outcomes) in terms of a childhood morbidity or psychomotor development in a cohort of offspring of GDM mothers vs. controls. The secondary aim was to (iii) explore whether mother´s obesity plays any role in an offspring development independently of GDM.

## Material and methods

The baseline study population comprised in total *n* = 432 participants, of those 364 had GDM and 68 had physiologic pregnancy according to old World Health Organisation (WHO) criteria [17]. Briefly, participants were recruited from several out-patient prenatal centres in the city of Brno, Czech Republic. All subjects participated in a routine GDM screening at mid-gestation (between 24-30^th^ week of pregnancy) performed by oral glucose tolerance test (oGTT) with 75 g of glucose. Given the fact that the recruitment of study subjects at baseline was ongoing before the International Association of Diabetes and Pregnancy Study Group (IADPSG) criteria were officially adopted in Czech Rep. but glucose values in 3-point oGTT were available, GDM diagnosis for the purpose of the recent study was defined according IADPSG criteria reclassification (fasting plasma glucose (FPG) ≥ 5.1 mmol/L, 1-h post-load glucose ≥ 10.0 mmol/L and 2-h post-load glucose ≥ 8.5 mmol/L with any one of the three cut-off values qualifying for the GDM diagnosis). Women diagnosed with GDM were followed from the time of GDM diagnosis till birth at the Diabetes Centre of the University Hospital Brno. The treatment for GDM included diet in all cases, whereby 27.9% of GDM cases required insulin therapy (there were no metformin users in the cohort). Exclusion criteria were T1DM or T2DM before pregnancy, non-Caucasian ethnicity, multiple pregnancies and severe comorbidities. More details on maternal characteristics of GDM and control population can be found in our previous publication [18].

All participants of the initial cohort (both GDM and healthy pregnant controls) delivered in the same hospital facility (Obstetrics and Gynaecology Clinics of the University Hospital Brno) and their electronic maternity records containing description of delivery and its event. complications and health status of the neonate until the discharge (such as gestational age, birth weight, Apgar score, presence of jaundice, etc.) were thus available. Maternal clinical and anthropometric characteristics are provided in the Table 1. Offspring perinatal data are shown in the Table 2. Following peripartal parameters were considered: ultrasonographic examination before delivery, a date of delivery, length of delivery, necessity of delivery induction, perinatal complications, post-delivery complications, section, abnormality of pH, base excess, Apgar score, birth weight.

**Table 1:** Clinical, anthropometric and biochemical data of mothers in the 2^nd^ trimester of pregnancy

Parameter	GDM (*n* = 26)	Controls (*n* = 63)	P	Parameter	Obese (*n* = 11)	Non-obese (*n* = 78)	P
Age (years)	33 (31–35)	31 (29–33)	NS	Age (years)	33 (31–34)	31 (29–34)	NS
Primipara	50%	54%	NS	Primipara	18.2%	58%	**0.018**
Pre-gestational BMI (kg/m^2^)	24.4 (22.7–27.7)	22.6 (20.0–24.8)	**0.019**	Pre-gestational BMI (kg/m^2^)	34.4 (30–34.9)	22.7 (20.1–24.4)	** < 1 × 10**^**–6**^
**Obesity (BMI above 30 kg/m**^**2**^**)**	23.1%	9.5%	NS	**GDM**	54.5%	25.6%	**0.048**
Diabetes mellitus in family anamnesis	81%	74.1%	NS	Diabetes mellitus in family anamnesis	82%	65.4%	NS
Stop-smoker	28.6%	7.8%	**0.012**	Stop-smoker	45.5%	11.5%	**0.004**
Breastfeeding (months)^a^	6 (3–22)	12 (8–17)	NS	Breastfeeding (months)	9.5 (6–22)	12 (6–17)	NS

Data expressed as a median (IQR) or proportions. Differences evaluated by nonparametric Mann–Whitney or chi-square test, respectively. ^a^Data about breastfeeding were evaluated in the questionary in 3 years of offspring age

*BMI* Body mass index, *GDM* Gestational diabetes mellitus

**Table 2:** Perinatal data

Parameter	GDM (*n* = 26)	Controls (*n* = 63)	P	Obese mothers (*n* = 11)	Non-obese mothers (*n* = 78)	P
Macrosomia (child birth weight above 4000 g)	7.7%	9.5%	NS	9.1%	9.0%	NS
Delivery induction (using oxytocin or Prostaglandin E)	27.8%	32.7%	NS	63.6%	18.0%	**0.0003**
Non-physiologic delivery (Caesarean section, VEX using, forceps using)	33.3%	14.6%	NS	9.1%	15.4%	NS
Abnormal length of delivery (too long = above 480 min, or too short = under 60 min)	27.8%	16.3%	NS	9.1%	15.4%	NS
Any adverse outcomes of offspring^a^	15.4%	9.5%	NS	0%	10.3%	NS
Complications after delivery (manual extraction of placenta, hypotonia uteri)	11.1%	0%	**0.019**	0%	2.6%	NS

Comparison was performed using chi-square test

*GDM* Gestational diabetes mellitus, *VEX* Vacuum extractor

^a^abnormal Apgar score (in 5^th^ min. < 5), abnormal base excess (< -12), abnormal cord blood pH (< 7.1), macrosomia

At the time of enrolment, a subset of *n* = 89 (20.6% of women at baseline) gave consent to participate in the prospective follow-up (by giving a written consent and providing contact details) and to periodically share data about their children development and health (see further). Of those *n* = 26 had GDM and *n* = 63 were controls. Couple of weeks before estimated delivery every child in Czech Republic is registered with primary care paediatrician and undergoes regular check-ups and vaccinations according to the unified nation-wide time schedule plus receives any eventual acute interventions and prescriptions for less serious illnesses or referral to specialised centres if necessary. Following the discharge from the maternity hospital the paediatrician will perform initial health check-up of the neonate day or two (up to one week) after delivery and is provided a copy of maternity record. The paediatrician then starts this own health record and issues a parent-held infant health record (PHIHR) containing concise summary of growth and development pattern, personal history of disease, immunisation history and other relevant data to parents. Further check-ups are scheduled at standardised intervals (at 3, 6, 12, 18 months following delivery and at 3, 5, 7 and bi-annually so forth up to 19 years of age, after which subjects are transferred to family physician). PHIHR contains fairly dichotomous data indicating whether development follows the expected progress or not (e.g. growth curves of infants who are exclusively breast-fed or formula fed for a given age, ability to perform standardised motoric tasks, expected onset of speech abilities etc. Gross deviations then require specialised appointments (physiotherapy, neurology, orthopaedics, ophthalmology, speech therapy etc.) with results and recommendations reported back in separate reports. The current study was designed as a pragmatic one relying solely on PHIHR information to retrieve time-standardised data for a given follow-up with the aim to provide a general overview of growth and developmental pattern in GDM and non-GDM offspring in a country with universally accessible GDM screening and high standard of care and to ascertain, whether any differences still exist to stress the health burden of GDM. All consenting study subjects willing to participate in the follow-up were periodically approached electronically by investigators—first by e-mails providing explanation of incoming procedure and then with the online link to the questionnaire (twice for each participant—spring 2017 and spring 2019). Questionnaire items corresponded with the parameters available from PHIHR to minimise errors when reported by parents. For the purpose of study following parameters were considered: weight, length/high, blood pressure, resting heart rate, duration of breastfeeding, psychomotor development (normal or abnormal, evaluated according to WHO recommendations for particular periods [19] including neuro-reflexes, sitting, standing, walking, keeping themselves clean = not wearing diapers…), status of senses (vision, hearing – normal or abnormal), status of nutrition (evaluated according to WHO recommendations [20] as malnutrition, overnutrition, food allergies etc.), status of school readiness (ready or not ready to start primary school education at age 6, evaluated by Czech recommendations [21] – i.e. colours recognition, drawing a person, determination a quantity, order, location), personal history of morbidity, need for regular drug prescription, need for a regular specialist follow-up and status of vaccination. Anthropometric data were used to construct a growth charts (The WHO Child Growth Standards weight-for-length, weight-for-height [22]).

Data were expressed as medians and interquartile ranges (IQR) or percentage for between-group comparisons. As the anthropometric data did not reveal a normal distribution (Kolmogorov–Smirnov test, *P* > 0.05), nonparametric tests were used in general for comparisons between and within the groups (Mann -Whitney and Wilcoxon tests, resp.). Chi-square test was used for contingency tables. Software Statistica (StatSoft, Tulsa, Oklahoma, USA) was used for all analyses. *P* < 0.05 was considered statistically significant.

## Results

GDM mothers had significantly higher BMI at the time of GDM diagnosis in the second trimester of pregnancy (*P* = 0.019, Mann–Whitney test). Prevalence of obesity was also higher in GDM group, but not statistically significantly. GDM mothers were more frequently stop-smokers (*P* = 0.012, Chi-square test). Controls breast-fed for longer periods (12 months, IQR 8 – 17) compared to GDM mothers (6 months, IQR 3 – 22), but the differences were not statistically significant (all *P* =  > 0.05, Mann–Whitney test). All parameters are included in the Table 1.

Perinatal parameters of both groups (GDM vs. controls) were in general comparable. GDM mothers had increased frequency of Caesarean section, instrumental delivery, abnormally long delivery (cut-off ≥ 480 min), worse offspring outcomes (namely lower Apgar score, base excess or cord blood pH) and more frequent complications after delivery, however, statistical significance was ascertained only for mother´s complications after delivery (need of manual extraction of placenta and/or uterine hypotonia, *P* = 0.019, Chi-square test). For all perinatal results see Table 2.

Comparison of PHIHR data at 12^th^ and 18^th^ months of age revealed significant differences in speech abilities with offspring of GDM mothers having worse outcomes in both time-points, namely inability to produce any word at 12^th^ and impaired word linking in 18^th^ months of age (*P* = 0.015 and *P* = 0.009 resp., Chi-square test). Speech abilities at 5 years of age were still better in the control group, but the difference was no more statistically significant. Psychomotor development and performance in school readiness test [21] at 5 years of age revealed borderline difference with worse outcomes in GDM group (both *P* = 0.048, Chi-square test). Offspring of GDM mothers were more frequent illness in their first 5 years of age and had more frequent need of hospitalisation (*P* = 0.022, Chi-square test) with upper respiratory tract infections and allergies as the most common causes. For all results see the Table 3.

**Table 3:** Offspring data

Parameter	GDM (*n* = 26)	Controls (*n* = 63)	P	Obese mothers (*n* = 11)	Non-obese mothers (*n* = 78)	P
Birth weight (g)	3405 (3010–3585)	3240 (3020–3600)	NS	3350 (3050–3540)	3270 (3000–3630)	NS
Newborn jaundice	12.5%	13.5%	NS	18.2%	10.3%	NS
Don´t say any word^a^	16.7%	1.9%	**0.015**	0%	6.4%	NS
Don´t link words^b^	45.8%	17.3%	**0.009**	45.5%	19.2%	**0.034**
Don´t walk alone^b^	8.7%	3.8%	NS	0%	5.1%	NS
Percentile weight-for-height ^c^	54 (33–63)	45 (23–65)	NS	56.5 (33–75)	46 (22–63)	NS
Percentile weight-for-height ^d^	57 (35–69)	37 (22–67)	NS	69 (62–85)	37 (22–65)	**0.04**
Systolic blood pressure (mmHg)^d^	95 (92–103)	100 (94–110)	NS	105 (95–110)	99 (92–105)	NS
Diastolic blood pressure (mmHg)^d^	52 (48–61)	58 (50–60)	NS	53 (51–58)	57.5 (50–60)	NS
Heart rate (/min)^d^	91 (80–98)	90 (81–103)	NS	69 (59–79)	91 (85–105)	NS
Abnormities in psychomotor development^d^	8.3%	0%	**0.048**	0%	1.3%	NS
Abnormities in nutritional status^d^	0%	2%	NS	0%	1.3%	NS
Abnormities in vision^d^	0%	2%	NS	0%	9%	NS
Abnormities in hearing^d^	0%	0%	NS	0%	0%	NS
Abnormities in speech^d^	30.8%	23.5%	NS	9.1%	19.2%	NS
Abnormities in school readiness test^d^	8.3%	0%	**0.048**	0%	1.3%	NS
Breastfeeding	87.5%	92.3%	NS	81.8%	76.9%	NS
Abnormities in vaccination	12.5%	13.5%	NS	0%	12.8%	NS
Need for regular specialist observation	44%	47.5%	NS	36.4%	46.2%	NS
Any illness/ hospitalisation	62.5%	23.9%	**0.022**	36.4%	29.5%	NS
Need for regular drug therapy	37.5%	16.4%	NS	18.1%	19.2%	NS

Data expressed as median [IQR], Mann–Whitney test, or frequency (%), chi-square test

^a^evaluated at 12 months of age, ^b^evaluated at 18 months of age, ^c^evaluated at 3 years of age, ^d^evaluated at 5 years of age

Experience of adverse perinatal outcomes (see Table 2) had no significant influence on offspring psychomotor development or morbidity up to 5 years of age (parameters from the Table 3) in any of the two groups (evaluated by Chi-square testing), however, results of this comparison are most probably significantly influenced by generally small number of children with perinatal adverse outcomes.

Smoking (or stop-smoking, *n* = 11 in the whole cohort) had no significant influence on perinatal outcomes in our study group, Children of (stop)smokers had slightly increased frequency of respiratory illnesses up to 5 years of age then those with non-smoking mothers (*P* = 0,048, chi square test), psychomotor development was not influenced by smoking in our cohort.

When we compared babies born with macrosomia (*n* = 8) vs. those with a normal birth weight (*n* = 81), regardless of GDM diagnosis of their mothers, children with macrosomia had a higher percentile of weight-for-height in 3 years (75, IQR [44 – 89] vs. 49 [24 – 64], *P* = NS) as well as in 5 years of age (67, IQR [51 – 91] vs. 42 [22 – 65], *P* = 0.024, Mann–Whitney test) and increased resting heart rate in 5 years (110, IQR [91 – 120] vs. 88, IQR [72 – 99], *P* = 0.05, Mann–Whitney test). Children with macrosomia were significantly more frequently ill up to 5 years of age compared to children with a normal birth weight (62.5% vs 23.9%, *P* = 0.022, Chi-square test).

As a secondary aim we compared data of obese vs. non-obese mothers, regardless of GDM diagnosis. Obese women (BMI before pregnancy ≥ 30, *n* = 11) reported significantly more stop-smoking (see the Table 1 – right side), more frequent need of an induced delivery (*P* = 0.0003, Chi-square test), for all perinatal data see Table 2, right side). Offspring of obese mothers had significantly worse verbal language skills in 18^th^ month of age (*P* = 0.034, Chi-square test), a higher percentile of weight-for-height in 3 years (*P* = NS) as well as in 5 years of age (*P* = 0.04, Mann–Whitney test). For all offspring data see Table 3, right side).

## Discussion

The aim of the current study was to prospectively follow offspring of GDM mothers vs. controls from their birth up to 5 years of age and to document event. differences in their psychomotor development and morbidity. The study is unique in including mother´s parameters during pregnancy together with perinatal data. Of note is the fact that the GDM cohort received the best possible prenatal and perinatal care and GDM was therefore well managed. As a secondary aim we compared offspring of obese vs. non-obese mothers, however, the validity of this analysis is diminished by small number of pre-pregnancy overtly obese respondents (*n* = 11). The fact that only 20% of enrolled women were willing to participate in the prospective part of study is no different from the results of other prospective studies focusing on GDM and reporting similar low compliance [23, 24].

Our main findings can be summarised as follows: GDM mothers have increased weight and more often were stop-smokers than those with a physiological pregnancy. Offspring of GDM mothers had delayed psychomotor development in early childhood but in 5 years of age they seemed to gradually achieve results of a control group. Children with macrosomia had a higher percentile of weight-for-height and were significantly more frequently ill than those with a normal birth weight. Offspring of obese mothers had worse verbal language skills in early childhood and a higher percentile of weight-for-height.

GDM mothers are at increased risk of adverse pregnancy outcomes, independently to BMI, especially to have offspring with macrosomia [25]. Most of the published studies described higher birth weight, increased occurrence of Caesarean sections, rarely a stillbirth in GDM group compared to controls [26–28]. Some studies indicate decreased breastfeeding in GDM mothers [29], however, the lower adherence to breastfeeding is probably caused by more frequent instrumental delivery, not the GDM diagnosis per se as confirmed by a big Australian study with a half a million of participants [30]. Results of our study generally copy the findings of previous studies, although the differences between GDM and control group were more commonly not statistically significant. The finding that GDM mothers had a higher BMI than controls and were significantly more often (stop)smokers (nobody admitted active smoking during pregnancy) had no significant influence on the perinatal parameters or offspring development in our cohort. However, previous studies detected that offspring exposed to smoking during pregnancy can have increased risk of severe mental illness [31] or later mental development [32].

Systematic review and meta-analysis monitoring psychomotor development of offspring of mothers with T1DM, T2DM or GDM showed the infants (at 1–2 years of age) of diabetic mothers had significantly lower scores of mental and psychomotor development compared to control infants, but results are based on observational cohorts and a direct causal influence of intrauterine hyperglycaemia remains uncertain [14]. The Polish study with a retrospective design included 59 children, 20 of them mothers with GDM, 19 of mothers with T1DM and 20 children of healthy mothers and found no abnormalities in a psychomotor development in offspring of GDM mothers [15]. We were able to find only single, 25 years old, prospective study focused on prenatal and perinatal influences on long-term psychomotor development in offspring of 196 diabetic mothers. They investigated antepartum maternal metabolism and proved a borderline association between the children's scores on the psychomotor development index at age 2 years and maternal third-trimester beta-hydroxybutyrate levels, no associations of altered psychomotor development with adverse perinatal outcomes or mother´s obesity were showed [16]. Results of our study show offspring of GDM mothers having significantly worse speech abilities in early childhood, but in 5 years of age they seem to gradually achieve results of a control group. The parturition played no role in psychomotor development in our study group, the same results are presented in the German study with 9,591 children which aimed at psychomotor development in the period between 3 and 7 years in relation to delivery and found no significant differences in the intelligence or any delayed motor development between instrumental or spontaneous delivery [33].

In terms of overall morbidity, our findings indicate increased morbidity in a cohort of offspring of GDM mothers compare to controls with respiratory tract illnesses and allergies being the most common. For example study of Kumar et al. [34] is rather consistent with our findings in reporting an increased occurrence of atopic status in early childhood of offspring of GDM mothers. The biologic mechanism by which GDM might affect susceptibility to atopic dermatitis and allergen sensitization remains unclear, however. For example, adiponectin attenuates allergic inflammation in murine models [35]. Thus, it is possible that altered levels of adipokines associated with GDM might have some effect on immunologic development in infancy.

Macrosomia itself can play an important role in an offspring life, previous epidemiological studies have shown that both low and high birth weight are associated with increased risks of obesity, cardiovascular disease and type 2 diabetes in later life [36, 37]. We confirmed a higher weight-for-height percentile in children with macrosomia and this percentile increased in time. Many studies analysed offspring macrosomia in conjunction with psychomotor development, they found that children born at over the 90^th^ percentile for weight have increased risk of attention deficit hyperactivity disorder (ADHD), depression, anxiety, autism, and cognitive delay (as summarised in [38]). Our findings indicate no difference in psychomotor development in macrosomic offspring compare to those with a normal birth weight.

As a secondary aim we focused on offspring of obese mothers in comparison with non-obese. Maternal obesity is in general associated with a greater risk of preterm birth, large-for-gestational-age babies, foetal defects, congenital anomalies and perinatal death [39]. Furthermore, breastfeeding initiation rates are lower and there is a greater risk of early breastfeeding cessation in women with obesity compared with healthy weight women [40]. In our study we detected a possible negative influence of mother´s obesity on speech abilities of their offspring in early childhood and similar results were described in a Spanish study [41] where maternal pre-pregnancy obesity was associated with a reduction in offspring verbal scores at pre-school age. A systematic review by Adane et al. [42] postulated that intrauterine environment has a detrimental effect on children’s cognitive development. However, evidence of the association between the maternal obesity and mental development of children is too scarce to offer a definite conclusion. We confirmed results of other studies by findings of higher percentiles weight-for-height in offspring of obese mothers, compare to non-obese, which can mean a higher risk of children obesity later in life [43, 44].

Given the prevalence of overweight/obesity is still much higher than that of GDM, focusing on pathophysiological mechanisms operating in obesity alone and obesity-GDM combination is extremely important. Animal models have shown that maternal diet-induced obesity is associated with elevations in inflammatory markers in the brain and morphological changes in hippocampal neurons with shorter and less numerous neurons [45]. Besides inflammation, other biological mechanisms through which maternal obesity can influence child neurodevelopment include leptin, a hormone produced by the adipose tissue that has been involved in mood disturbances, thyroid dysfunction, nutrient deficits such as vitamin D or folic acid, and neurotoxins cumulated in the adipose tissue (i.e. mercury, persistent organic pollutants etc.) [46]. The foetal environment in overt maternal diabetes (preceding pregnancy) is mainly characterized by hyperglycaemia, chronic hypoxia and iron deficiency, complemented with recurrent acute changes in glucose status and academia. Moreover, pregnancy altered glycaemia may affect foetal development, have a significant impact on offspring cognition, and also increase the risk of suffering from mental disorder [14]. One might expect similar effects operating in GDM pregnancy, although to lesser degree compared to T1DM.

One of the potential limitations of our study with a possible influence on the quality and the interpretation of the findings could be the fact, that all GDM subjects were followed and all study participants (GDM and controls) delivered in a tertiary health care centre, so they obtain the best possible care and our findings might not be universally representative. Furthermore, we also did not account for a possible effect of paternal obesity or other morbidities (incl. diabetes) in our analyses potentially influencing offspring results in the study as well. Moreover, results of analyses focusing on children with macrosomia or offspring of overtly obese women in our study could be distorted by a small number of cases. Yet, our findings still confirm results of other studies. Finally, the biggest limitation seems to be a disproportion between the numbers of participants with GDM vs. controls willing to be prospectively followed from the baseline study group. However, comparison of the baseline (summarised the Table 1) as well as the perinatal data (summarised in the Table 2) of participants of followed in the prospective study vs. remaining subjects in GDM and control groups did not reveal any statistically differences. We therefore assume that any eventual selection bias is minimal. The reasons for low compliance of patients with GDM (unwillingness to participate in the follow up studies) might only be speculated incl. managing the new disease, a new role as mother, lack of motivation to contribute to research, privacy concerns etc. Several studies confirm our theory [47–49].

## Conclusions

In conclusion, we present here a unique prospective study focusing on psychomotor development of offspring of GDM and non-GDM mothers from birth up to 5 years of age. Despite the very good diabetes control and prenatal care we revealed several differences in selected parameters in offspring of GDM mothers, mainly in speech abilities and total morbidity. Moreover, a significant link of mother´s obesity and offspring increased adiposity was detectable in our cohort, but, because of relatively small number of obese participants in our study, we cannot reliably separate those effects. Maternal gestational diabetes and obesity can be therefore considered an important determinant of postnatal offspring development and health status, which further advocates for broader implementation of preventive strategies aiming at obesity and GDM in women of reproductive age.

## Data Availability

The datasets generated the current study are not publicly available but are available from the corresponding author on reasonable request.

## Abbreviations

ADHD: Attention deficit hyperactivity disorder
BMI: Body mass index
CVD: Cardiovascular disease
FPG: Fasting plasma glucose
GDM: Gestational diabetes mellitus
IADPSG: International association of diabetes and pregnancy study group
IQR: Interquartile range
oGTT: Oral glucose tolerance test
PHIHR: Parent-held infant health record
T1DM: Type 1 diabetes mellitus
T2DM: Type 2 diabetes mellitus
WHO: World health organisation
